# Volcanic unrest as seen from the magmatic source: Reyðarártindur pluton, Iceland

**DOI:** 10.1038/s41598-023-50880-0

**Published:** 2024-01-10

**Authors:** Emma Rhodes, Steffi Burchardt, Sonja H. M. Greiner, Tobias Mattsson, Freysteinn Sigmundsson, Tobias Schmiedel, Abigail K. Barker, Taylor Witcher

**Affiliations:** 1 Department of Earth Sciences, Uppsala University, 754 36 Uppsala, Sweden; 2 Center for Natural Hazards and Disaster Science, Uppsala University, 754 36 Uppsala, Sweden; 3 Nordvulk, Institute of Earth Sciences, University of Iceland, 101 Reykjavík, Iceland; 4 School of Earth and Environmental Sciences, University of St Andrews, St Andrews, KY16 9AJ Scotland; 5 Department of Geological Sciences, Stockholm University, 106 91 Stockholm, Sweden; 6 Department Geoscience and Engineering, TU Delft, 2628 CN Delft, The Netherlands

**Keywords:** Volcanology, Natural hazards

## Abstract

How the Earth’s crust accommodates magma emplacement influences the signals that can be detected by monitoring volcano seismicity and surface deformation, which are routinely used to forecast volcanic eruptions. However, we lack direct observational links between deformation caused by magma emplacement and monitoring signals. Here we use field mapping and photogrammetry to quantify deformation caused by the emplacement of at least 2.5 km^3^ of silicic magma in the Reyðarártindur pluton, Southeast Iceland. Our results show that magma emplacement triggered minor and local roof uplift, and that magma reservoir growth was largely aseismic by piecemeal floor subsidence. The occurrence and arrangement of fractures and faults in the reservoir roof can be explained by magmatic overpressure, suggesting that magma influx was not fully accommodated by floor subsidence. The tensile and shear fracturing would have caused detectable seismicity. Overpressure eventually culminated in eruption, as evidenced by exposed conduits that are associated with pronounced local subsidence of the roof rocks, corresponding to the formation of an asymmetric graben at the volcano surface. Hence, the field observations highlight processes that may take place within silicic volcanoes, not accounted for in widely used models to interpret volcanic unrest.

## Introduction

**Figure 1:**
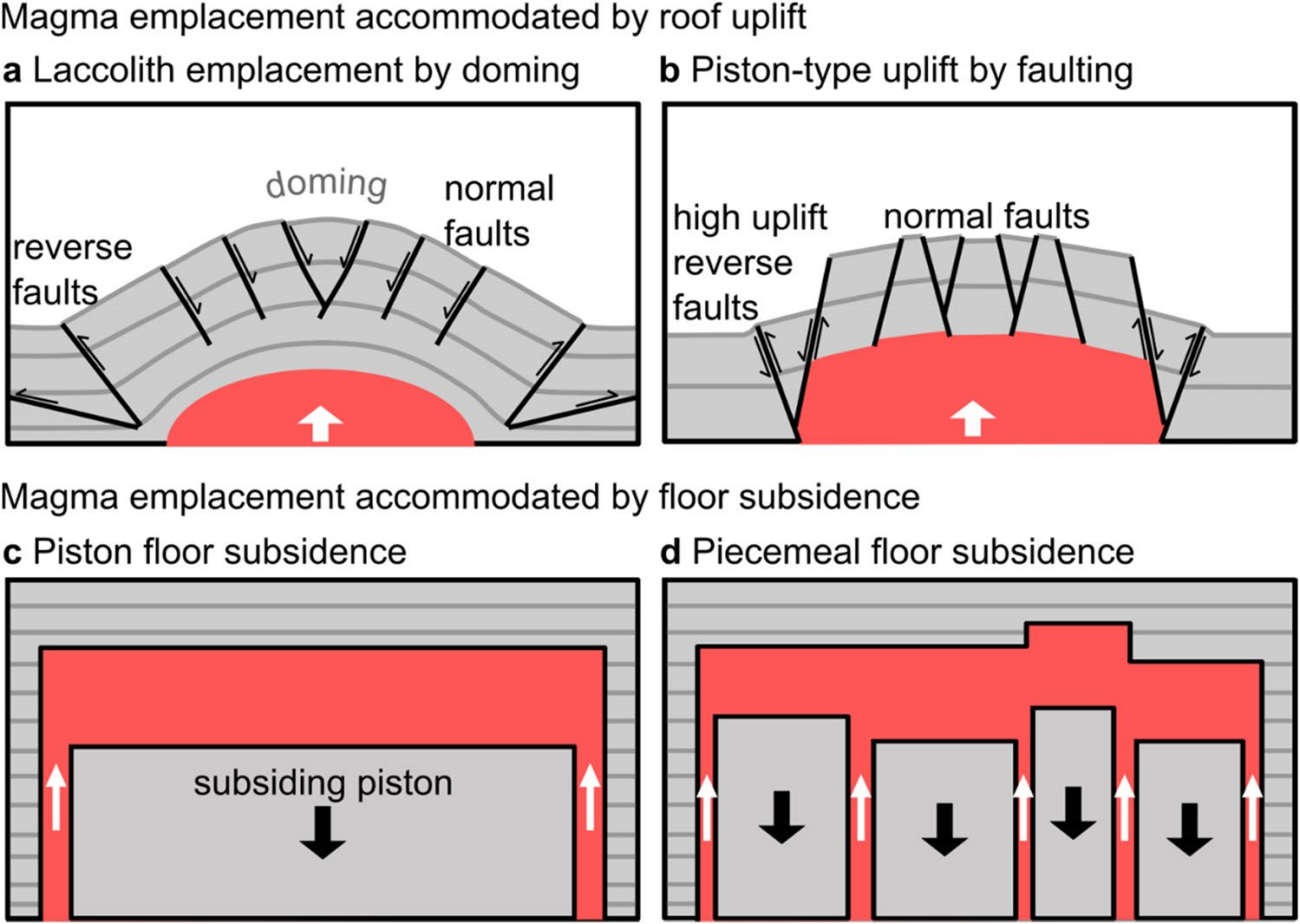
Primary emplacement models for magma reservoirs in the mid-upper crust. (**a**,**b**) Magma emplacement accommodated by roof uplift. (**a**) Roof doming typically produces a ‘forced fold’ in the host rock with sets of moderately dipping normal faults above the centre. Shallow dipping thrusts flank the intrusion (cf. refs.^17,18^). (**b**) In the piston-uplift scenario, the host rock should largely retain its original inclination, and large steeply dipping faults that facilitate uplift should be visible at the edges of the intrusion^19–22^. Figure adapted from Schmiedel et al*.* (2019). (**c**,**d**) Magma emplacement by floor subsidence. (**c**) The traditional floor subsidence model is via the detachment and subsidence of a single piston of rock, with a roof geometry defined by the first intrusion of magma (in this example, horizontal). Magma is fed to the growing reservoir via ‘ring dykes’ that surround the subsiding block^4–6,23^. (**d**) Piecemeal floor subsidence is similar to piston subsidence, but occurs via multiple floor blocks bound by faults and multiple source dykes^24,25^.

New magma ascending beneath active volcanoes requires the creation of space, which is primarily facilitated by deformation of the surrounding host rock. In the mid to upper crust, space for magma is accommodated by two mechanisms (Fig. 1): (1) the uplift of the host rock above the intruding magma (the magma reservoir ‘roof’) via laccolith emplacement^1–3^; and (2) the subsidence of the rocks below the intruding magma (the magma reservoir ‘floor’)^4–8^. The type of emplacement mechanism influences the ground deformation and seismic signals that can be detected via volcano monitoring equipment^9^. The capacity for space to be created both initially, and during the growth of a magma reservoir, also influences the potential for overpressure build-up within the magma reservoir and therefore a potential eruption^10^. Nowadays, surface deformation and seismicity prior to, and during eruptions, is monitored in unprecedented detail. However, the interpretation of these signals relies on our understanding of how magma migrates and is stored within the crust^9,11,12^. Reconstruction of the geometry of solidified magma reservoirs (also called plutons) and observations of structures indicating host-rock deformation offer a way to infer magma emplacement mechanisms^13–16^. Furthermore, the structures in the roof of a pluton may record the formation of conduits that fed eruptions^15^.

Here, we use the Reyðarártindur pluton in Southeast Iceland as a case study to explore how space was created for the formation of a reservoir of silicic magma. Secondly, we investigate the deformation of the host rock associated with eruption, and conditions that led to eruption. To do this, we use field mapping and photogrammetry to analyse the orientation of the lava layers and fractures, faults and dykes in the host rock to the pluton. We then combine this information with the 3D pluton shape reconstruction from Rhodes et al. (2021) and suggest that the Reyðarártindur pluton was emplaced predominantly via floor subsidence. We show that the brittle roof structures were created by overpressure in the pluton rather than by regional tectonics and link the overpressure build-up and eruption potential to floor subsidence failure. In order to quantify what, if any, deformation would be expected at the Earth’s surface during eruption, we constructed a simple numerical model that replicates the field observations of subsidence towards one of the conduits. Finally, we discuss the likely detectable signals related to the emplacement, growth, and eruption of magma reservoirs comparable to Reyðarártindur. With this case study, we aim to improve the interpretation of geophysical signals and creation of models for periods of magma movement and volcanic unrest in shallow magmatic systems.

### Geological setting

Volcanism in Iceland is caused by the Iceland mantle plume and the divergent Mid-Atlantic Ridge (MAR)^26^. Active volcanism occurs along (1) rift zones that coincide with the plate boundary and (2) flank or off-rift zones, which are not plate boundaries^27–29^. Rift zone segments are connected by WNW–ESE transform fault zones, such as the Tjörnes Transform Zone and the South Iceland Seismic Zone^30^. Within the rift zones, volcanism occurs in individual volcanic systems, which contain fissure swarms and (often) a central volcano^31^. Volcanic systems generally have a NNE–SSW trend, perpendicular to the spreading direction of the MAR, and parallel to the fissure swarms that consist of normal faults, extensional fractures and volcanic fissures^32,33^.

**Figure 2:**
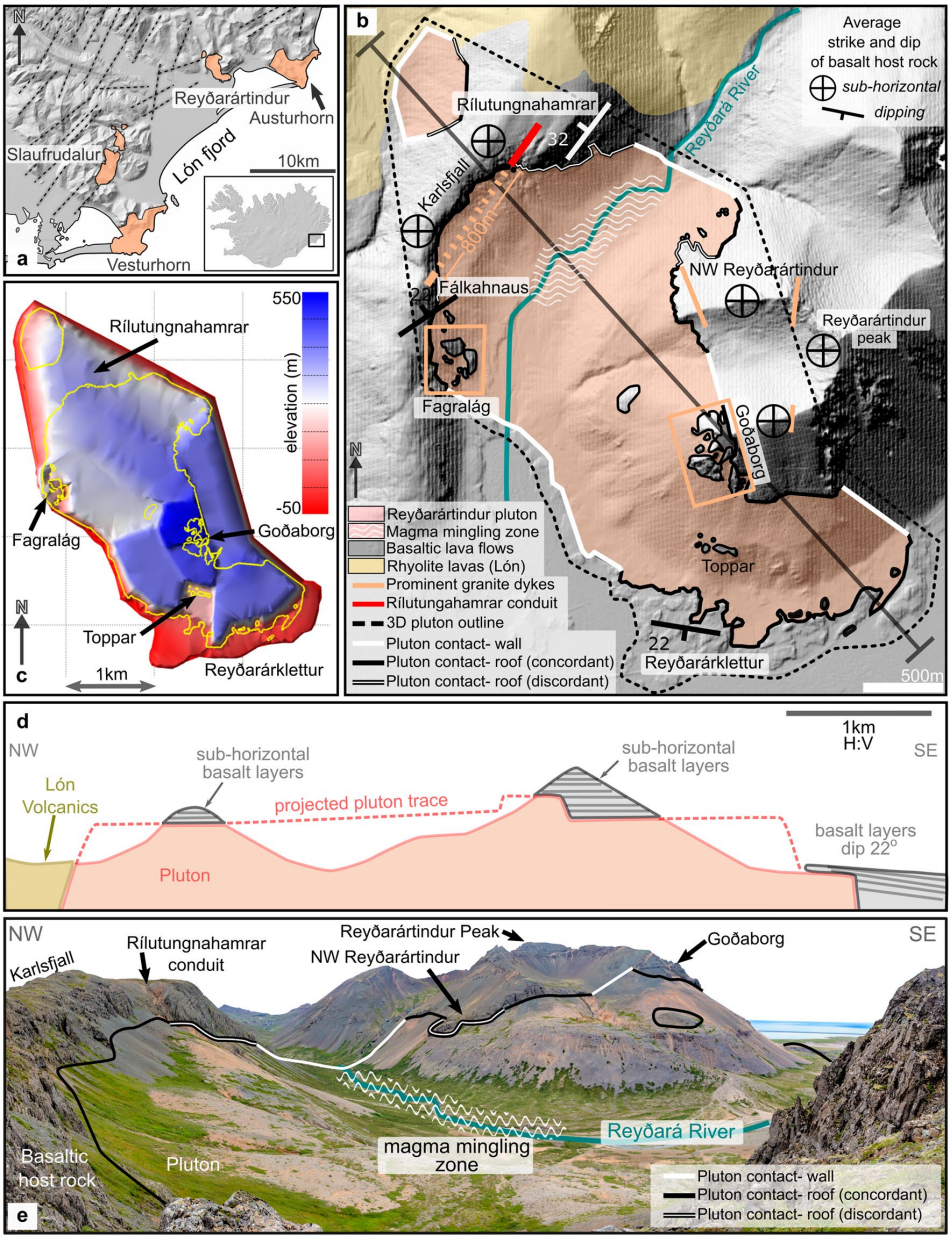
Background information and overview of the Reyðarártindur Pluton. (**a**) Map of the plutons exposed within the Lón fjord of Southeast Iceland, with generalized trends of basaltic dyke swarms (dashed lines) collated by Walker 1974. The major dyke trends are a proxy for the former rift axis. Pluton outlines are based on maps produced by refs.^13,15,36,39^. Inset: Map of Iceland with location of Lón fjord. (**b**) Map of the Reyðarártindur pluton showing the exposed contact, average strike and dip of the host rock, prominent granite dykes, and Rílutungnahamrar conduit. Site names have been adapted from the Landmælingar Íslands map viewer (www.lmi.is). (**c**) 3D shape reconstruction of the pluton to − 50 m asl in aerial view, modified after Rhodes et al*.* (2021). The floor of the pluton is not exposed, therefore a minimum lower elevation of − 50 m asl was inferred based on outcrop exposure at sea level. The mapped contact is shown in yellow. (**d**) Cross section NW–SE through the pluton. Cross section trace marked in (**b**). (**e**) Overview photo of the Reyðarártindur Pluton, looking eastwards from Fálkahnaus. Maps a, b and c created in MOVE 2019.1 software (https://www.petex.com/products/move-suite/).

The Reyðarártindur pluton is exposed in the mountains surrounding the Lón fjord in Southeast Iceland (ref.^34^; Fig. 2a). The geology of Lón is characterised by the juxtaposition of a number of Neogene volcanic systems, which comprise volcanic rocks deposited in and around central volcanoes. Dyke swarms representing the subsurface feeders of volcanic fissures mostly strike NNE–SSW and NE–SW, indicating the direction of the rift zone at the time of dyke emplacement^34^. Moreover, kilometre-sized silicic and mafic-silicic plutonic complexes crosscut the volcanic deposits. One of these intrusions is the Reyðarártindur pluton, which yields zircon crystallization ages of 7.40 Ma^35^. The plutons likely formed at a depth of 1–2 km beneath a paleo rift-zone^36–38^ and are regarded as the solidified magma reservoirs that fed eruptions in younger central volcanoes^15,34,38^.

## Results

### Results from previous studies

The Reyðarártindur pluton was emplaced into sub-horizontal basaltic lava flows of Neogene age, and in the north-west of the study area, rhyolite lavas of the Lón Volcano (Fig. 2b). Mapping of the exposed pluton and its host rock by Rhodes et al. (2021) documented that adjacent roof exposures occur with vertical offsets of up to 200 m, which creates structural highs and lows. The 3D reconstruction of the pluton shape by Rhodes et al. (2021) shows a complex angular rhomboid with a long axis trending NW–SE, with steps in the roof and a minimum volume of 2.5 km^3^. Minor changes to the pluton outline and 3D reconstruction were made in this study (Fig. 2c). Analysis of the internal magmatic lithology showed that the pluton is mainly constructed from a single rock unit, the Main Granite^15^. Local zones of mingling between the Main Granite and two other related magmas (quartz monzonite to granite) are exposed in the Reyðará River zone (Fig. 2b). Furthermore, Rhodes et al*.* (2021) identified that the pluton also fed eruptions from three locations; Rílutungnahamrar, Fagralág and Goðaborg (Fig. 2b,c,e). While the paleo-surface is not exposed, evidence for eruptive activity was based on the exposure of prominent dykes originating from the pluton. These dykes contain rocks with pyroclastic, brecciated and tuffisitic textures and are associated with local subsidence. Additionally, the same magmatic rock units as in the Reyðará River zone are exposed within the Fagralág and Rílutungnahamrar conduits.

### Observations and orientations of the host rock and the roof contacts

**Figure 3:**
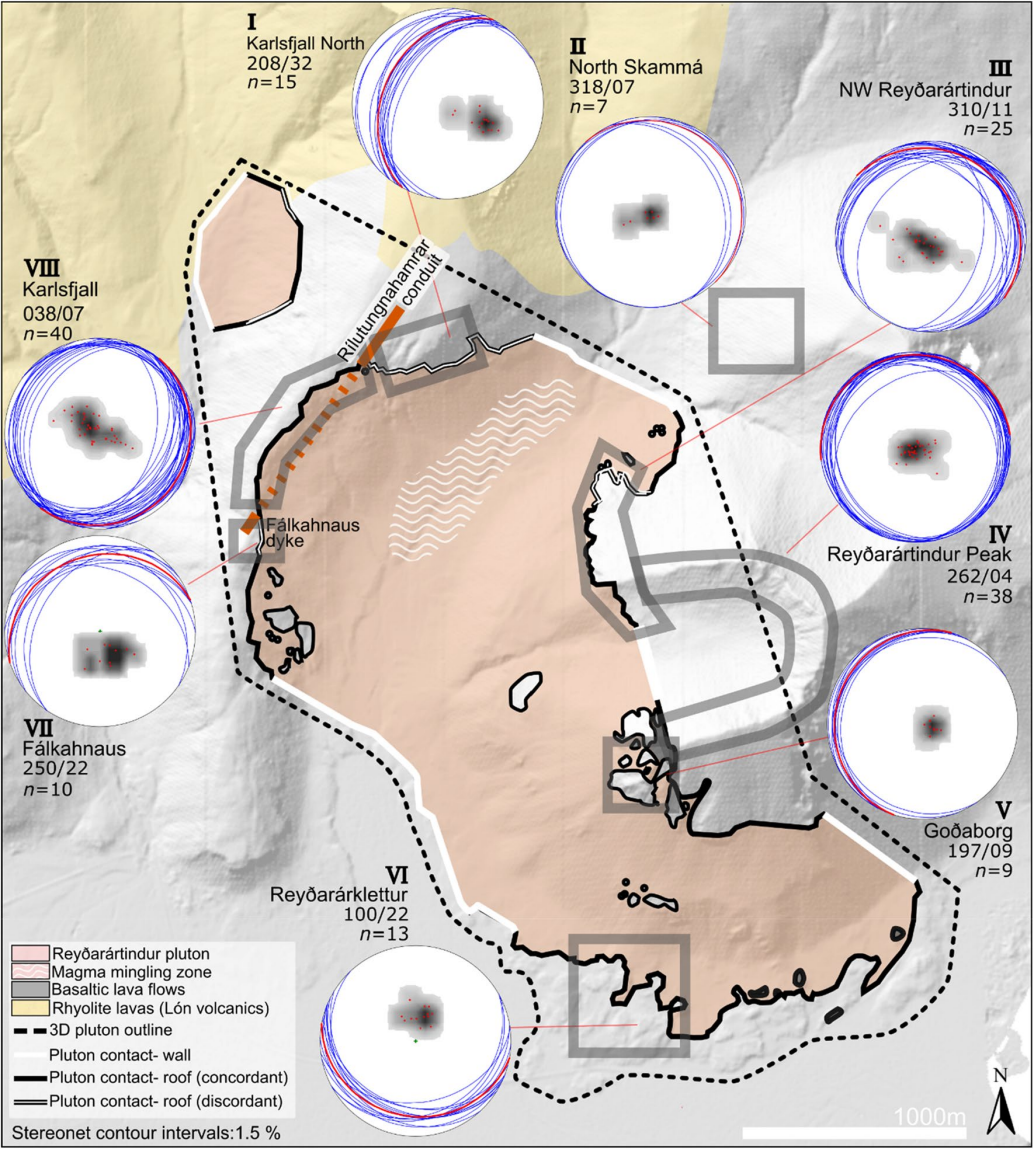
Orientation of bedding in the host lavas to the Reyðarártindur Pluton. Planes and poles to bedding are displayed in Schmidt stereonet plots (equal area, lower hemisphere). Red plane indicates the mean lava orientation, which is additionally reported in strike/dip convention beside the relevant stereonet. *n* denotes the number of measurements in the stereonet plot. The outline of the Reyðarártindur Pluton is adapted from Rhodes et al*.* (2021). Map created in MOVE 2019.1 software (https://www.petex.com/products/move-suite/).

We quantified host rock deformation by mapping of lava orientations in the host rock. Our measurements of the lavas at a distance of 500 m from the pluton contact (North Skammá) show northerly dips of less than 7° (Fig. 3: stereonet II), providing a background for comparison with lava orientations near the pluton.

**Figure 4:**
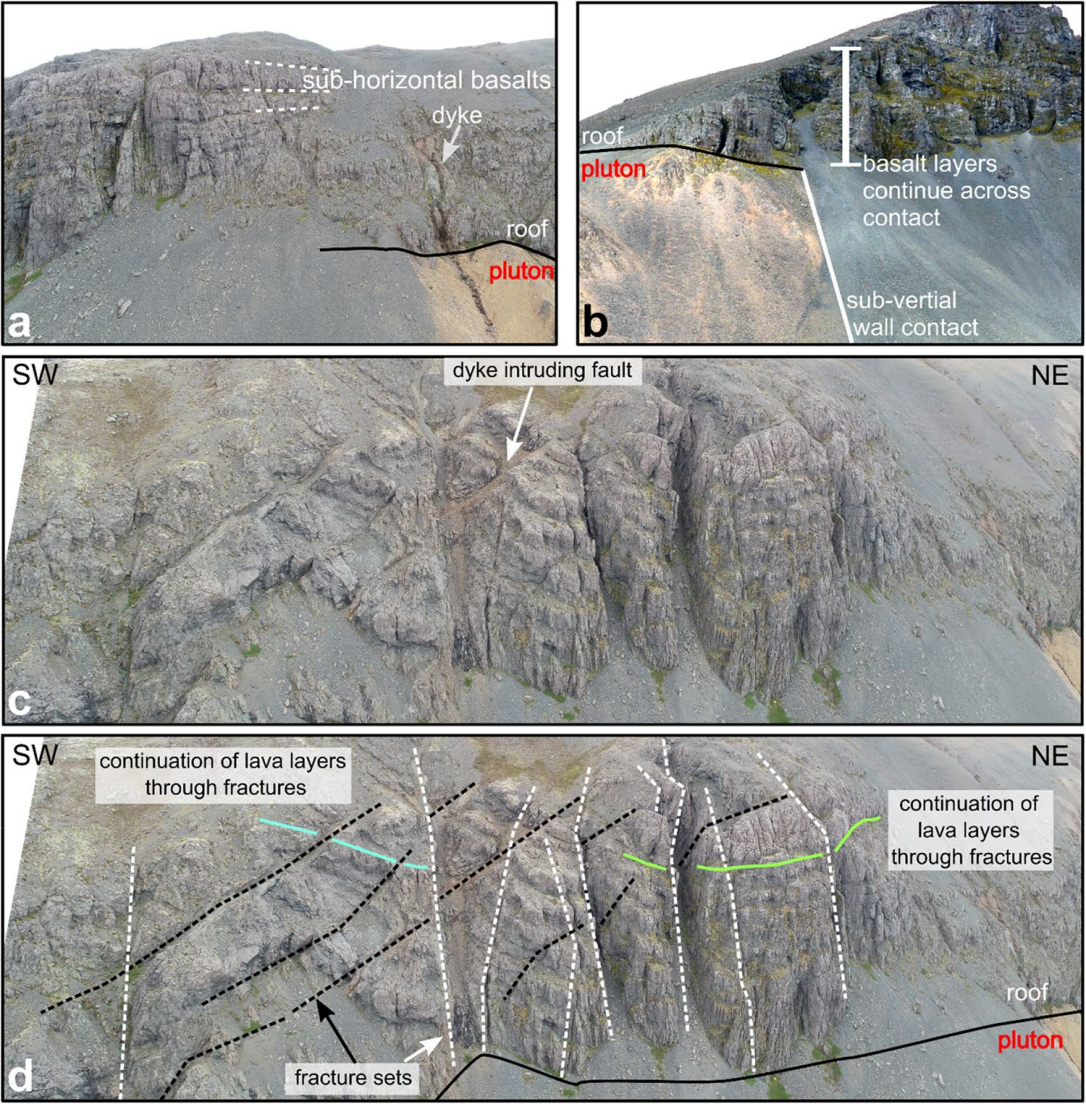
Photos of host rock features to the Reyðarártindur Pluton. (**a**) A typical horizontal roof contact with conformable basalt lava flows. (**b**) Example of a sub-vertical wall contact where the conformable basalts continue across the contact. Unfortunately, the wall sides and the (inferred) underlying basalt are covered by scree. (**c**, **d**) Unmanned Aerial Vehicle (UAV) photo, and interpretation of features in a roof section exposed along Karlsfjall ridge. Two fault sets are clearly visible at this site. Lava layers can be traced across faults and dykes with up to 5 m offset.

Likewise, our measurements of the lavas above the pluton are generally sub-horizontal (0°–12°), although dip directions vary from site to site, and locally between faults (Figs. 3, 4). While the roof contact is usually concordant with the layering of the overlying basalts, a few discordant contacts occur (e.g. at NW Reyðarártindur; Fig. 2b). Large vertical offsets of the pluton roof of up to 200 m create both structural highs (e.g. at Goðaborg) and lows (e.g., at Toppar), resulting in a ‘stepped’ pluton roof contact in 3D (Fig. 2c). Notably, the magmatic rocks of the pluton do not vary or show evidence of faulting in the vicinity of the steps in the pluton roof, which rules out that the steps are the result of tectonic faulting after the pluton solidified. Sparse outcrops and the contact trace suggest that the pluton wall contacts are sub-vertical and discordant to the host lavas (Fig. 4b). The lava beds at the wall contacts are sub-horizontal, e.g., as measured within the Reyðarártindur peak dataset (Fig. 3: stereonet IV).

**Figure 5:**
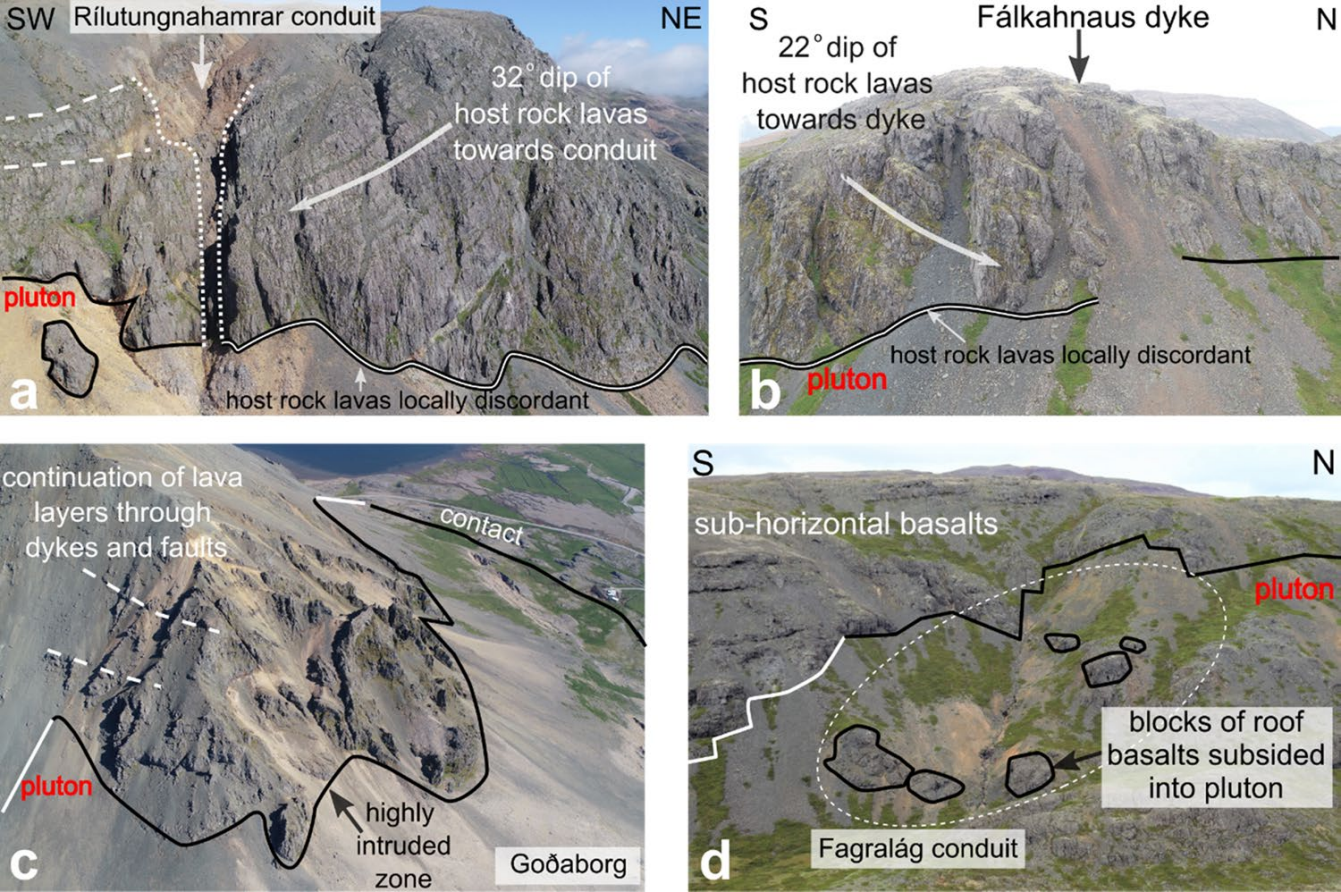
Key features of dykes and conduits exposed at Reyðarártindur (**a**) UAV photo of Rílutungnahamrar. On the SW side of the conduit, the host rock lavas are sub-horizontal, whereas on the NE side they dip towards the conduit at ca. 32°, and the roof contact is locally discordant to the pluton contact. The conduit widens upwards. (**b**) UAV photo of the Fálkahnaus dyke. On the south side, the lava layers are discordant to the pluton contact and locally dip ca. 22° towards the dyke. (**c**) UAV photo looking eastwards down on Goðaborg, where the roof is heavily intruded by dykes from the Reyðarártindur pluton. Lava layers are continuous across dykes and faults. (**d**) UAV photo of the Fagralág locality, where blocks of the downfaulted roof are exposed as isolated blocks.

We identified three dip anomalies of the lava layers in the pluton roof. The first dip anomaly occurs at Reyðarárklettur where the lavas locally dip ca. 22° to the south (Fig. 3: stereonet VI). The second dip anomaly occurs to the east of the Rílutungnahamrar conduit. Here the lava layers dip at ca. 32° NE towards the conduit, and are locally discordant at the pluton contact (Figs. 3: stereonet I; 5a). In contrast, to the west of the conduit at the locality labelled Karlsfjall, the lavas are mostly sub-horizontal (0°–10°: Figs. 3: stereonet VIII, 5a), although affected by faults (see below). Based on the 32° dip of lava layering, which extends ca. 250 m from the conduit in cross-sectional view, we estimate that the pluton roof east of the conduit has subsided by 295 m, while no or insignificant subsidence occurred west of the conduit. The third dip anomaly occurs at the locality labelled Fálkahnaus on Fig. 3. Here, a 20 m wide, 60° striking dyke we refer to as the Fálkahnaus dyke is exposed in the host rock (Fig. 5b), and the lavas on the southern side of the dyke dip up to 22° to the NNW, i.e., roughly towards the dyke (Figs. 3: stereonet VII, 5b). The 22° dip anomaly continues for 180 m to the south, thereby yielding 65 m of subsidence at the dyke plane. The Fálkahnaus dyke is exposed in along-strike prolongation from the Rílutungnahamrar dyke, leading us to conclude that they are connected (i.e. as per the orange dashed line on Fig. 3).

### Observations and orientations of fractures, faults and dykes in the host rock

**Figure 6:**
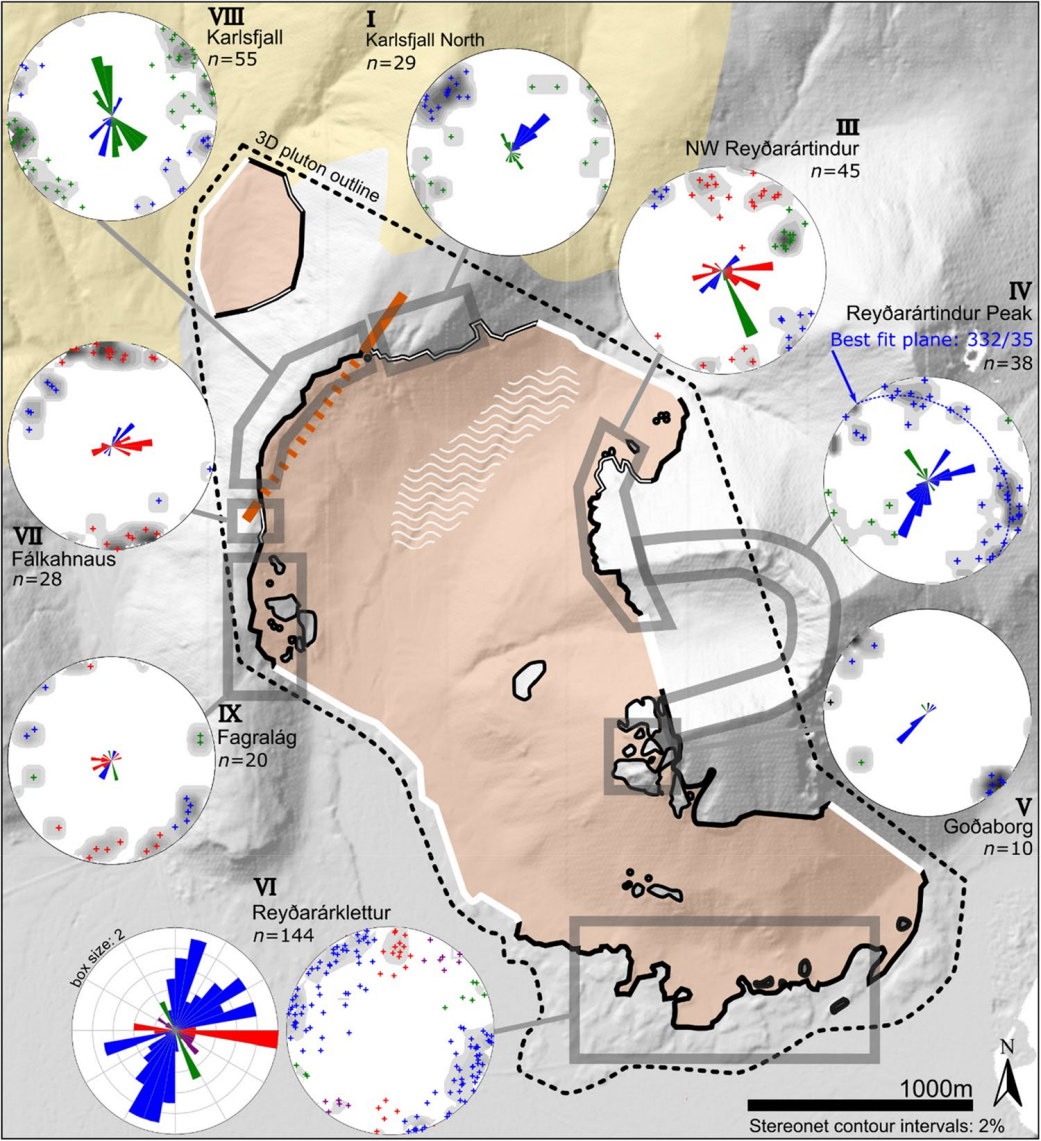
Orientation of fractures, faults and dykes (FFDs) in the host lavas to the Reyðarártindur pluton. Poles to planes of FFDs are displayed in Schmidt stereonet plots (equal area, lower hemisphere). The colours represent the different FFD sets described in the text. Rose plots (bidirectional, linear scaling, class size 10°) display the strike of the FFDs and are scaled to the Reyðarárklettur dataset. For the pluton outline legend, refer to Fig. 4. Map created in MOVE 2019.1 software (https://www.petex.com/products/move-suite/).

The basaltic lavas overlying the pluton roof are fractured, faulted and intruded by granitic dykes that extend upwards, and thus likely originate from, the pluton. This is in contrast to the site at North Skammá away from the pluton, which does not exhibit these features (Fig. 2b). Near the pluton roof, the fractures are steeply dipping to sub-vertical (70°–90°) and some of the fractures exhibit sub-vertical displacement of the lava layers of up to 5 m (i.e. they are faults) (Figs. 3, 6). Dykes are 0.5–10 m wide, follow fractures, and can be widely spaced (ca. 100 m between dykes) or occur in densely spaced (ca. 5 m) clusters, mimicking the distribution of fractures. One particular dyke cluster is associated with the highest topographical step in the pluton roof at Goðaborg peak, a locality that is also highly fractured (Fig. 5c). Another dyke-and-fracture cluster (80 m wide, NE–SW striking) can be traced through the ridgelines of Reyðarártindur peak and may be associated with the eastern pluton wall contact (cf. Fig. 2b).

Most dykes occupy fractures and faults, and dyke identification is often obscured by scree. Thus, fracture, fault and dyke (FFD) orientations were not measured separately. The results are displayed in Fig. 6 and show that multiple FFD sets occur across the pluton roof, which can be described as follows for the specific areas:

- NW Reyðarártindur displays three FFD sets (stereonet III). The first set is sub-vertical with polymodal distribution, and strikes NE–SW. The second set is also sub-vertical with polymodal distribution and strikes approximately E–W. The third FFD set is SE striking, SW dipping with normal displacement.
- Reyðarártindur Peak also displays two FFD sets (stereonet IV). The first set, which is most dominant, has a radial pattern with a best-fit plane of 332/35. The second set is conjugate with subvertical orientation and strikes NW–SE.
- At Goðaborg Peak (stereonet V), one primary FFD set is exposed which is steeply dipping and conjugate with NE–SW orientation. Two further individual faults were measured with N–S and NW–SE orientations (black and green poles to planes: Fig. 6).
- At Karlsfjall North (stereonet I), where the lavas dip NW towards the Rílutungnahamrar conduit, two FFD sets are displayed. The first FFD set is polymodal and strikes NW–SE, and the second strikes NE and shows normal displacement. Displacement of up to 2 m was measured along both these FFD’s, with progressive subsidence to the north.
- Karlsfjall (stereonet VIII) displays polymodal FFD sets oriented NW–SE and NE–SW. Alternatively, the first set could also be interpreted as quadrimodal, with sets oriented 135°–315° (NNW–SSE) and 170°–350° (NW–SE).
- At Fálkahnaus (stereonet VII), where the lavas dip NNW towards the Fálkahnaus dyke, polymodal FFD sets were measured oriented E–W and NE–SW.
- The Fagralág conduit site (stereonet IX) displays polymodal FFD sets oriented NE–SW, and approximately E–W. A conjugate set was additionally measured oriented NW–SE.
- At Reyðarárklettur (stereonet VI), FFD orientations can be divided into (a) a major polymodal set oriented N–NE/S–SW with a strong cluster at 0°–30°/180°–210°, (b) a major conjugate set oriented E–W, and (c) other minor conjugate sets oriented NW–SE and WNW–ESE, which may be quadrimodal or polymodal to the E–W set.

In summary, the FFD sets measured show conjugate or polymodal distributions with orthorhombic symmetry^40,41^ (Fig. 6). The specific density and orientation of FFDs vary from site to site, but three main FFD sets are consistently measured which strike NE–SW, NW–SE, and E–W. Which of these FFD sets are represented at individual locations varies slightly, but the absence of one or more sets can generally be explained by sampling bias due to the shape of the outcrop.

## Discussion

The structures preserved at Reyðarártindur represent the sum of successive processes encompassing the establishment and growth of a magma reservoir, as well as eruption, and cooling. In the following, we will discuss which deformation features can be assigned to what stage in the pluton evolution. Then we infer what volcano monitoring signals would have corresponded to the deformation during each stage.

### Initial magma emplacement

Generally, the shape of plutons, the relationship with primary host-rock structures, and structures related to emplacement deformation are proxies for the type of magma emplacement^13,17,42^. In the case of the Reyðarártindur pluton, distinguishing emplacement by either roof uplift or floor subsidence should account for:

1. its rhomboid shape with highs and lows in the roof, concordant sub-horizontal roof and discordant steep wall contacts (Figs. 2, 3, 4),
2. the distribution of intrusive rocks inside the pluton with mingling in the Reyðarártindur River zone and the conduits (ref.^15^; Fig. 2b),
3. the absence of significant uplift or tilting of the overlying host rocks except in the Reyðarárklettur locality (Fig. 3, stereonet VI) and the continuity of roof rocks beyond the pluton boundaries (Fig. 4b), and
4. the existence, distribution, and orientation of multiple FFD sets above the pluton roof (Fig. 6).

**Figure 7:**
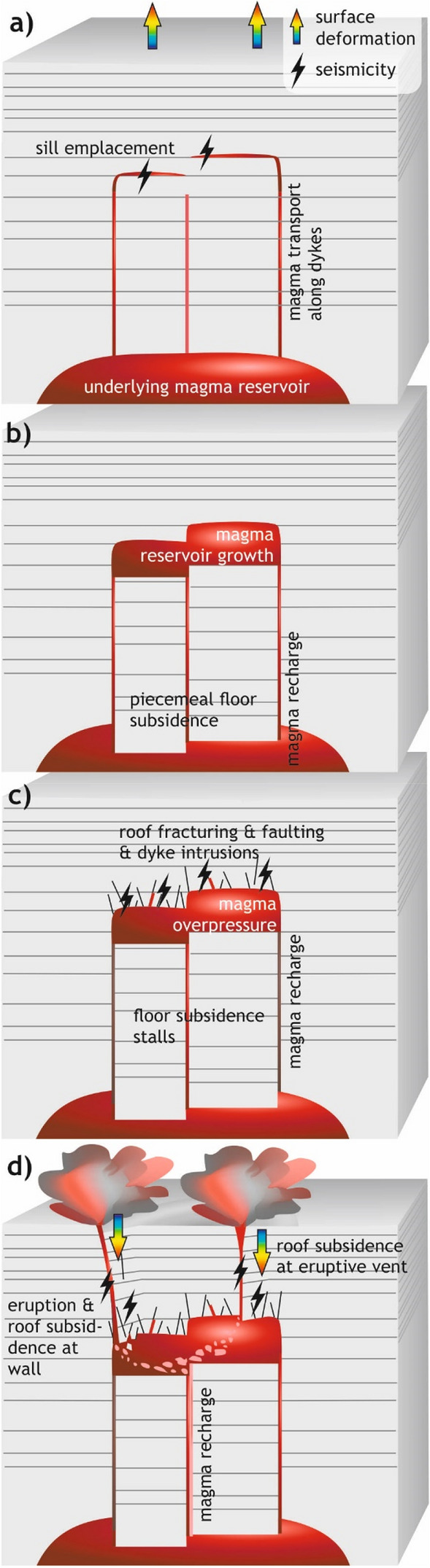
Conceptual model for the emplacement to eruption of the Reyðarártindur pluton which links the geological observations and shows the corresponding volcanic unrest signals. (**a**) Magma ascends from the source reservoir via multiple dykes and is emplaced as sills at different depths. The upward propagation of dykes would likely be recorded in terms of seismicity following the trace of the propagating dykes and characteristic ‘trough and bulge’ surface deformation. Sill emplacement would cause characteristic surface uplift and minor seismicity during propagation. (**b**) Blocks of rock, dislodged by the dykes and sills subside into the underlying magma reservoir. Magma is transferred from there into the growing Reyðarártindur magma reservoir. As long as the subsidence of the upper reservoir can accommodate the magma transfer, this stage is aseismic and does not create surface deformation. (**c**) When floor subsidence stalls, magma recharge creates overpressure in the Reyðarártindur magma reservoir. This creates fault and fracture sets in the reservoir roof, some of which get intruded by dykes. Roof fracturing and faulting would have caused detectable minor earthquakes across the pluton roof. (**d**) Recharge of magma with slightly different composition caused dyking at several locations in the reservoir roof. Subsequent eruption at the Earth’s surface and associated magma withdrawal caused significant, local subsidence of the reservoir roof, which was likely seismogenic.

These observations demonstrate that roof uplift did not cause the emplacement of > 2.5 km^3^ of magma. Only the minor (22° S) local tilt of the pluton roof in the south (Figs. 2, 3) may have been caused by roof uplift or uneven floor subsidence during magma emplacement. Moreover, the steep, discordant wall rocks support magma emplacement by floor subsidence along steeply-dipping ring faults/dykes (cf. refs.^5,6,24,25,43^). Traditional floor subsidence models include the subsidence of a single piston of rock with an intrusion roof defined by a singular either bell-shaped or a horizontal surface, defined by the first intrusion of magma^4–6,44^. At Reyðarártindur, however, we observe a stepped, horizontal roof, with offsets of 100’s of metres (Fig. 2c,d). The internal continuity of the magmatic rock, as well as the absence of faults with large displacements in the roof rocks point to the roof steps as primary, emplacement-related features (cf. ref.^24^). Consequently, we interpret the stepped roof as evidence that magma emplacement initiated simultaneously at several nearby localities corresponding to the roof steps (Fig. 7a). The resulting piecemeal subsidence implies that multiple blocks of the pluton floor subsided into the underlying magma reservoir, and magma was transferred between the blocks from the lower to the upper reservoir^24,25^. Continued magma supply would have promoted the thickening and subsequent merging of individual intrusions (Fig. 7b)^24,25^.

### Deformation during continued magma reservoir growth

Magma emplacement by floor subsidence does, however, not explain the distribution and orientation of brittle deformation features (FFDs) in the roof rocks of the Reyðarártindur pluton, nor do tectonic stresses in the rift zone. As indicated by the orientation of regional dyke swarms (ref.^34^; Fig. 2a), rifting during the Neogene likely occurred in NNE–SSW and NE–SW striking volcanic zones and would have produced consistently oriented sets of extension fractures with this strike^45–47^. Indeed, NNE–SSW and NE–SW striking FFD sets occur in the roof of the Reyðarártindur pluton (Fig. 6) and may indicate the influence of the concurrent tectonic stress field (Fig. 2a). However, FFD sets with wide ranges of orientations are measured across the pluton roof, with polymodal fracture sets additionally measured striking NW–SE and E–W (Fig. 6). Furthermore, and locally, normal faulting and radial fault patterns occur (NW Reyðarártindur and Reyðarártindur Peak; Fig. 6: stereonet IV). Hence, we consider the orientation of FFDs reflect the existence of a local, magmatic stress field juxtaposed on the rift-related tectonic stresses^40^.

Specifically, we consider that local stress fields created by magma pressure likely dominated during FFD formation, and that they attest to periods of overpressure in the magma reservoir. Polymodal fault sets point to the interaction between the local stress field created by the magma body and the regional stress field^48^. This is in agreement with other pluton studies that have attributed bimodal or quadrimodal fractures to pressure from magma exerted on the magma-chamber roof^13^. Alternatively, the FFDs may reflect preferential reactivation of a specific, pre-existing fracture set^14^. The fractures and faults provided pathways for magma as indicated by the numerous dykes, which follow the brittle roof discontinuities (refs.^48,49^; Fig. 4). Moreover, stress concentrations at the sharp roof-wall transitions of the magma reservoir may explain the increased density of FFDs at locations such at the wall contact (e.g. Reyðarártindur peak; cf. ref^50^; Fig. 2b). The build-up of magmatic overpressure implies that subsidence of the pluton floor was insufficient in accommodating subsequent magma recharge. We envisage that cooling and sealing of the faults and magma pathways between the underlying magma source and the Reyðarártindur magma reservoir inhibited continued subsidence of the reservoir floor (Fig. 7c).

### Transition from reservoir growth to eruption

Once the overpressure was sufficient to allow dyke propagation all the way to the Earth’s surface, an eruption could occur (cf. refs.^10,51^). The mingling of magmatic units with compositional ranges from quartz monzonite to granite within the Rílutungnahamrar and Fagralág conduits suggests that injection of new magma into the reservoir triggered eruption^15^. The locations of the three conduits in the roof of the pluton show that eruptions originated from (1) a structural high in the centre (Goðaborg), (2) at the roof-wall transition (Fagralág), and (3) from dykes cutting across the roof (Rílutungnahamrar–Fálkahnaus; Fig. 7d). The NE–SW strike of the Rílutungnahamrar–Fálkahnaus conduit suggests that the dyke geometry and eruption location was likely controlled by regional tectonics or occurred along a pre-existing, tectonic weakness (cf. Fig. 2). In contrast, the eruption of magma at the other two localities may have been related to stress concentration at steps in the roof (Goðaborg), or at a roof-wall transition (Fagralág)^50^. Hence, eruption locations and configurations reflect the interplay between the local magmatic stress field, the regional tectonic stress field, as well as the deformation features produced during magma reservoir emplacement and growth. Moreover, since both the Fagralág and the Rílutungnahamrar–Fálkahnaus conduits contain rocks equivalent to the mingled magmatic suite in the Reyðará River, and since Fagralág is located adjacent to Fálkahnaus, we may speculate that the eruptions from both conduits were contemporaneous and related.

Our mapping of the lava layering and FFDs in the pluton roof identified pronounced, local subsidence of the magma reservoir roof spatially associated with the Fagralág and Rílutungnahamrar–Fálkahnaus conduits. The type of subsidence observed at these locations is unlike that found at the structural highs and lows elsewhere in the pluton roof, where (1) roof layering is continuous, (2) the roof contact is mostly concordant, and (3) the rocks in the dykes show no evidence of explosive brecciation.

At Fagralág, multiple blocks of the roof subsided vertically, ‘piecemeal-style’ up to 150 m into the magma reservoir (ref.^15^; Fig. 5d). Notably, no lava dip anomalies were observed in this zone. The pre-existing fractures and faults in the pluton roof were likely used as planes of weakness for dyke intrusion, and facilitated subsidence of the roof blocks. Reconstruction of the volume of subsidence at Fagralág as a rectangular prism with an average depth of 100 m (from mapped roof blocks), width and length of 300 m (area exposed in map view), gives a volume of 9 million cubic metres (Supplementary Material 1). This number should correspond to the minimum amount of magma erupted minus the volume of magma remaining in the conduit.

At Rílutungnahamrar–Fálkahnaus, subsidence occurred in an asymmetric, trapdoor-like manner, flanked by a dyke that is exposed in both locations and widens upwards at Rílutungnahamrar. Tilting of the roof lavas by 20°–30° in a NW direction towards the dyke, and some additional reactivation of conjugate faults added up to between 65 and 295 m of subsidence of the previously flat reservoir roof (100 m on average). The trapdoor subsidence likely affected the entire width of the roof of Reyðarártindur, although with higher rates of subsidence at Rílutungnahamrar. Evidence for this is the continuation of the tilted lavas at Rílutungnahamrar all the way from the dyke exposure to the northern pluton wall contact (Fig. 2b). Hence, at the Rílutungnahamrar–Fálkahnaus conduit the minimum volume of magma erupted was 19 million cubic metres_,_ (as calculated from the triangular prism formed by a conduit length of 1300 m, a subsidence width in map view of 250 m and lava dip of 25°; Supplementary Material 1).

### Volcanic unrest signals related to magma emplacement and eruption at Reyðarártindur

Our conceptual model of the emplacement and eruption of magma at Reyðarártindur can be linked to volcanic unrest signals that would be recorded by monitoring at active volcanoes. Surface deformation and seismicity following the upwards propagation of dykes would likely be recorded (e.g. cf. refs.^52–54^; Fig. 7a). Seismic and deformation signals would then have changed as magma propagated laterally parallel to the host rock lava layers (Fig. 7a). Since in this stage magma propagates along pre-existing weaknesses in the rock (e.g. the contact surface between lava flows)^55^, seismicity may have been at a lower magnitude compared to the dyke-propagation stage. Broad surface uplift such as observed during sill formation^9^ may have been recorded early on, before floor subsidence was fully established. However, the scale of the surface uplift would significantly underestimate the volume of the intruding reservoir, as most of the magma emplacement was accommodated for by the downward-displacement of the floor along the subvertical feeders lubricated by magma. Hence, further growth of the magma reservoir by floor subsidence would have likely been aseismic and without any significant surface deformation (Fig. 7b). Aseismic magma chamber recharge has been documented for example at Colli Albani, Italy, and Cordon Caulle, Chile, instead inferred from ground inflation^56,57^, which was minimal at Reyðarártindur. If a comparable process is operating at an active volcano, it may be hard to detect by volcano monitoring systems. Magma can thus accumulate at shallow depths inside volcanoes without producing signals easily detectable by volcano monitoring equipment.

Following the establishment of the Reyðarártindur magma reservoir by floor subsidence, there were three post-emplacement processes that would have created detectable deformation and/or seismicity. Firstly, the build-up of overpressure in the chamber led to roof fracturing and faulting and/or fracture reactivation across the entire reservoir roof (Figs. 4,6,7c). Additionally, it may have produced the local tilting of the roof observed at Reyðarárklettur (Fig. 4). While the former would have likely been detected in terms of minor earthquakes, likely across the entire pluton roof, the latter would have caused slight localised surface deformation. Secondly, the propagation of the dykes into the chamber roof, and in some cases to the surface, would have likely caused both seismicity and surface deformation (as discussed above; Fig. 7d). Finally, the eruption of magma from at least three locations at the crest, the edge, and across the chamber roof would have been picked up by volcano monitoring (Fig. 7d).

**Figure 8:**
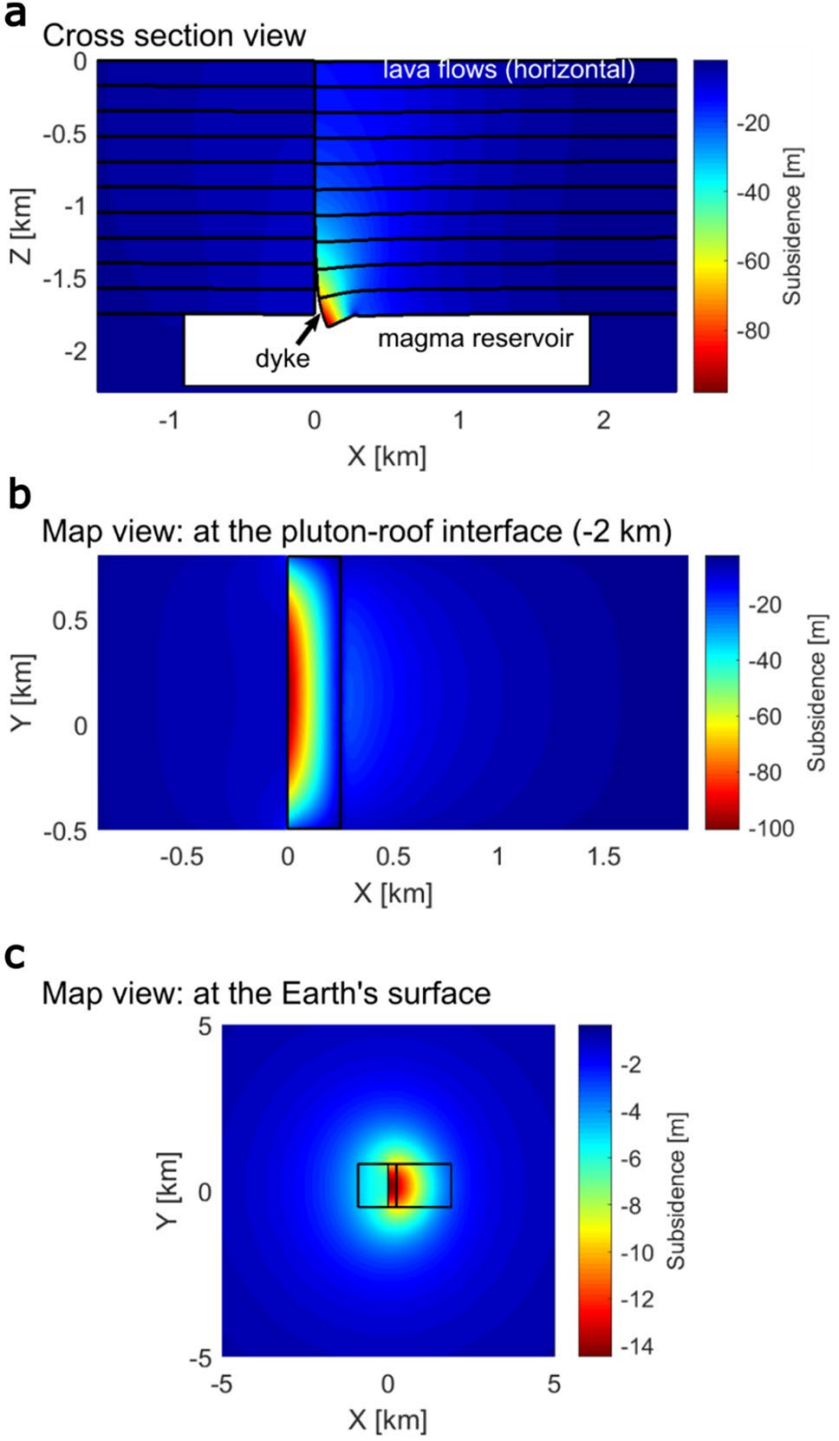
Results of the COMSOL Multiphysics deformation model simulating the effects of the observed roof subsidence on the east side of the Rílutungnahamrar–Fálkahnaus conduit. (**a**) X–Z cross section. Subsidence is localized to a zone close to the dyke. Black lines indicate the tilt of previously horizontal lines (i.e. lava layers). (**b**) Subsidence of the pluton roof at the pluton roof interface. The black box marks the zone which was forced to subside. The dyke is located at the left edge of the black box. (**c**) Subsidence observed at the Earth’s surface (i.e. 1.75 km above the pluton roof). The black box indicates the pluton extents in the model.

In order to simulate the surface deformation associated with the trapdoor subsidence during the Rílutungnahamrar–Fálkahnaus eruption, we implemented a 3D finite-element model in COMSOL Multiphysics®. The model results (Fig. 8) show that vertical subsidence of 100 m at the dyke centre can reproduce the ca. 25° lava dip by pure tilting, and the left side of the dyke is little affected by the forced subsidence. Because of the subsidence, the dyke is widest at the base and narrows towards the surface, which is in contrast to the upward-widening observed in the field (Fig. 5). However, the present-day geometry of the Rílutungnahamrar conduit is the result of post-diking processes, such as the establishment and evolution of a vent (e.g. ref.^58^). At the model surface, a half-graben structure is produced which has a length corresponding to the dyke length and a maximum depth closest to the dyke. Due to the linear-elastic properties of the host rock, the 100 m subsidence at the magma chamber roof only partially translates to the surface, where the maximum surface displacement is ca. 15% of the maximum subsidence at 1.75 km depth (Fig. 8). Interestingly, the surface deformation pattern is highly asymmetric and concentrates above the collapsing part of the roof, not the dyke. Subsidence of the magma-reservoir roof may have released significant seismic energy, especially if the subsidence occurred *en mass*^59^. The field observations highlight localised and unsymmetrical deformation with respect to the location of eruptive vents. This suggests faulting is important to consider when interpreting volcano deformation patterns, rather than the use of the homogeneous uniform elastic halfspace commonly used to interpret volcano deformation^60^.

## Conclusions

The emplacement of silicic magma at Reyðarártindur was accommodated by piecemeal floor subsidence. While initial magma chamber emplacement would have likely been detectable via seismic and geodetic monitoring, reservoir growth may have been aseismic. Magma overpressure caused small-scale faulting and fracturing of the reservoir roof, and eventually led to at least one eruption. This eruption occurred from a fissure with an orientation consistent with the regional-tectonic setting. Simultaneously, the eruption was associated with localised roof subsidence, which would have been observable at the Earth’s surface and may have caused significant seismicity. Hence, the study highlights processes that can take place in the volcanic plumbing system, not accounted for in widely used models to interpret volcanic unrest.

## Methods

### Structural orientation data

Structural orientation data of FFDs and lava beds were collected both in the field, and from virtual outcrops generated by structure-from-motion photogrammetry. In the field, the structural orientations were acquired using (a) the FieldMove Clino Pro application (www.mve.com/digital-mapping/) on two different Iphone 6® phones in the coordinate system UTM Zone 28N, or (b) analogue compasses accompanied by a Garmin GPSMAP handheld GPS. The Fieldmove Clino Pro application automatically corrected the Iphone measurements for the magnetic declination (–9.01), and the compasses were manually corrected prior to use.

Structure-from-motion photogrammetry was performed on 9 areas in total. Overlapping photos of the zones were acquired using a DJI Phantom 4 Pro UAV (Unmanned Aerial Vehicle) with a photo resolution of 5472 × 3648 pixels. The images were then processed to create virtual outcrops using the default workflow in the Agisoft Photoscan™ software (www.agisoft.com/). The internal GPS of the UAV was used for georeferencing, and low-quality model inputs were reduced using the “Estimate image quality”, “Reconstruction uncertainty” and “Projection accuracy” functions. The resulting .obj virtual outcrop was imported into the LIME v2.0 software (https://virtualoutcrop.com/lime/; ref.^61^), where the ‘structural data from 3 points’ tool was used to acquire the orientation of measurable FFDs and lava bedding. The tool can best constrain the orientation when the feature of measurement intersects 3D topography, i.e., a lava bed traces through a gully or around a ridge. In the case that the feature did not, we omitted to measure it. For this reason, the dips of many lava beds were not measured.

After data acquisition, all the measurements were collated in the Petroleum Experts MOVE 2019.1 software, where we plotted and analysed the data in equal-area stereographic projections of the lower hemisphere.

### Map outline and pluton shape reconstruction

The map outline and hence 3D pluton reconstruction were updated from Rhodes et al., 2021. Additional field mapping in the Steinasel area (Fig. 2) led to a revision of the pluton wall contact. Specifically, the wall contact at ca. − 1,645,000, 9,467,000 changes orientation from NNE–SSW to strike NNW–SSW, and now connects directly to the corner at Fagralág. The 3D pluton reconstruction was modified accordingly in the MOVE software and the change in volume was negligible (< 0.02 km^3^).

### COMSOL modelling of host rock subsidence around Rílutungnahamrar–Fálkahnaus dyke.

A Finite Element Model (FEM) was constructed using COMSOL Multiphysics® version 5.5 (www.comsol.com) which reproduces the ca. 25° tilt of the roof basalts towards the eastern side of the Rílutungnahamrar–Fálkahnaus dyke (Figs. 3, 5). The model assumes that the lava layers were sub-horizontal prior to dyke emplacement, which is consistent with the other field observations of the host rock (Fig. 4).

The model domain is assumed to consist of a linearly elastic, homogeneous and isotropic material with a Young’s modulus of E = 30 GPa and a Poisson’s ratio of ν = 0.25 cf.^62^. A domain measuring 250 × 250 × 250 km was used to avoid any edge effects. The magma body was modelled as a cavity (e.g., refs.^63–65^) centred at 2 km depth with a box shape measuring 2.8 × 1.3 × 0.5 km^15,36,37^. To model the Rílutungnahamrar–Fálkahnaus dyke, the model domain was sliced in the Y direction by a plane. Where the dyke was modelled on this plane the model domain contacts are defined as disconnected (“contact pairs”). The dyke spans the short-axis of the magma body and extends from the pluton roof at 1.75 km depth to the surface of the model (Supplementary Material 2). For the remainder of the plane the contacts were modelled as connected (“identity boundary pairs”). The surface of the model domain is allowed to deform freely, while the base is fixed, and boundary-parallel motion is allowed at the sides of the model domain.

A 250 m–wide part of the pluton roof adjacent to the right side of the dyke is forced to subside. The subsidence is a linear function of the distance to the dyke (x–direction) and a parabolic function of the distance from the edges of the pluton along the dyke (y–direction):

1 $$d\left( {x,y} \right) = \left[ {{\text{tan}}\left( a \right)\left( {x - 250m} \right) + b} \right] \cdot \left[ {\frac{{b - c}}{{{\text{400}},{\text{000}}m^{2} c}}y^{2} - 0.375\frac{{b - c}}{{500mc}}y + 1} \right]$$

where *a* describes the observed tilt of the lava layers (here we used *a* = 25°)*, b* is the subsidence of the roof at the edges of the part which is forced to subside (apart from the edge at the dyke) and *c* = *−* *tan(a)∙250 m* + *b* and corresponds to the subsidence at the Rílutungnahamrar outcrop (located at x,y = 0,0). The first term of Eq. (1) describes linear subsidence of a 250 m wide part of the roof towards the dyke (largest deformation directly at the dyke and decreasing with increasing distance). The second term lets the subsidence vary with y in a way so that the subsided roof has the shape of a parabola in a yz-cross section. The term is normalized to ensure that it is equal to 1 at the Rílutungnahamrar-outcrop. It is important to note that this function only influenced the displacement in the z–direction. Deformation in the horizontal was not specified. Expanded methods are presented in Supplementary Material 3.

### Supplementary Information

Supplementary Information.

## Data Availability

The datasets generated and analysed during the current study available from the corresponding author on reasonable request or can be downloaded from 10.5281/zenodo.10428597.
