# Supplementary material for: Volcanic unrest as seen from the magmatic source: Reyðarártindur pluton, Iceland
Source: Sci Rep. 2024 Jan 10;14:962. doi: 10.1038/s41598-023-50880-0 (PMC10781678; doi:10.1038/s41598-023-50880-0)
Supplement: Supplementary file 1 — Supplementary Information. [file 41598_2023_50880_MOESM1_ESM.docx]

## Supplementary Material


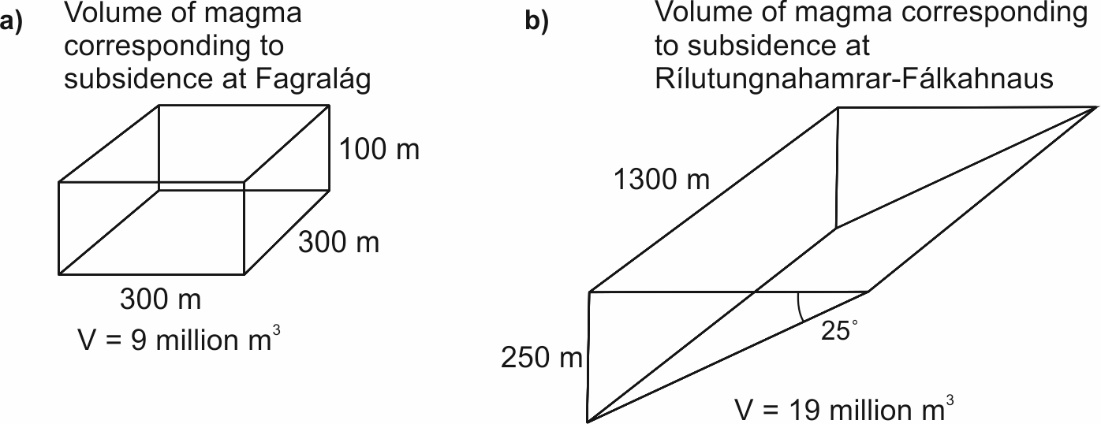


Supplementary Material 1: Sketches of the dimensions of subsided roof portions in the pluton, a) Fagralág and b) Rílutungnahamrar-Fálkahnaus. These dimensions are constrained by mapping in the field and through photogrammetry and form the base of volume calculations.


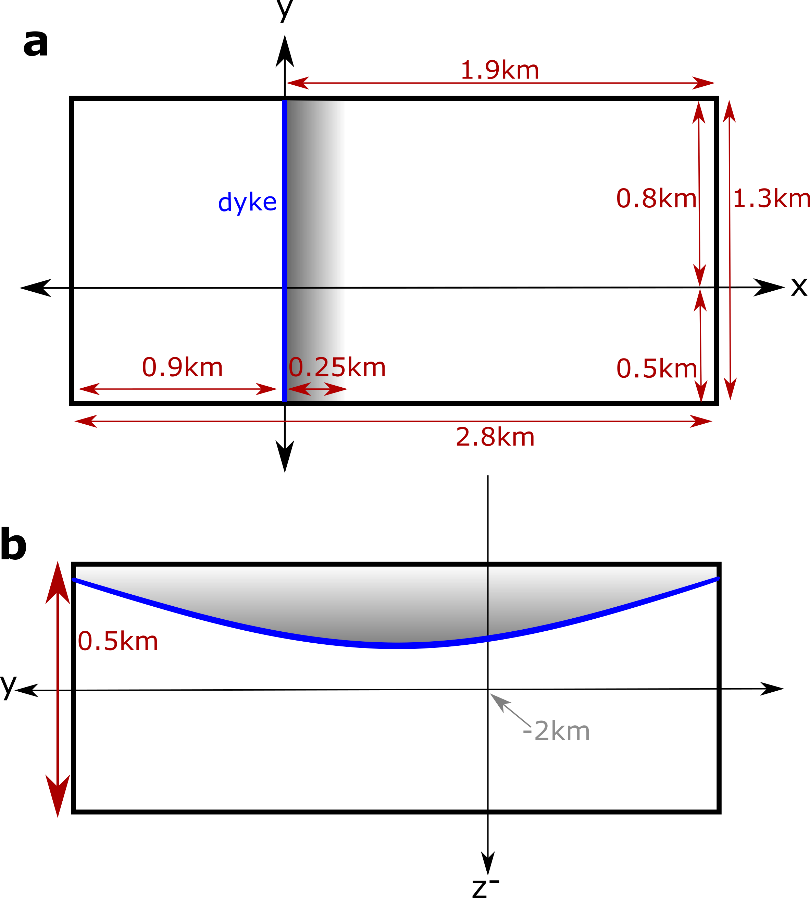


**Supplementary Material 2:** **Geometry of the modelled magma body as seen from the top and the side.** **(a)** Map view of the magma body. The dyke is marked in blue and the subsiding part of the roof is marked in grey with a darker colour indicating stronger subsidence. (**b)** Y-Z-cross section through the magma body (looking from the position of the dyke into positive X-direction). Z is defined as positive upwards. The blue line indicates the shape of the roof after the subsidence, which is highest in the middle, and lowest at the pluton edges.

### **Supplementary Material 3: Finite-element modelling of magma chamber roof subsidence**

A Finite-element deformation model was included into this study to obtain a first-order estimate of the surface deformation associated with the processes suggested for the Reyðarártindur pluton and to test, if a simple model can reproduce the observed asymmetric tilt of the overlying lava layers. It is important to note here that the model is strongly simplified and is used here as a qualitative tool to gather information about the overall pattern of expected surface deformation rather than to obtain precise quantitative estimates. A physics-driven, more realistic model of the pluton roof subsiding in connection with a dyke would have been beyond the scope of this study.

The model was implemented using the solid-mechanics module of the Finite element software COMSOL Multiphysics 5.5 and focuses on the purely elastic reaction of the host rock to pressure changes in the magma reservoir and to the opening of the dyke. Fluid flow within the magma body and the opened dyke as well as in-elastic deformation are not considered here.

The dimensions of the modelled magma body and connected dyke are described in the main text and shown in Figure S2. The entire domain is assumed to behave as a linear elastic material with a Young’s modulus E=30 GPa and a Poisson ratio ν=0.25. Pre-existing stresses, e.g. due to plate spreading are not considered here even though they likely influenced the process to some degree. The base of the model domain was set to be fixed entirely and no boundary-normal displacement was allowed at the lateral model boundaries (“Roller condition”). The entire model domain measured 250 x 250 x 250 km and the lateral boundaries were real, i.e. no infinite-element conditions were applied. The top surface of the model remained unconstraint.

The following section describes the derivation of the forced displacement applied to the 250 m-wide section of the pluton roof east of the dyke. It was modelled to vary in both x- and y-direction independently and can be written as

(2)

$$d\left( x,y \right)=f(x)\cdot g(y)$$

The function f(x) is linear and the slope should have an angle of a=25° to the horizontal. Since the subsiding part of the roof is 250 m wide, the maximum subsidence is equal to c’=-tan(a)∙250m. To ensure, that the subsidence is equal to c’ at x=0, the function is shifted to the right side

(3)

$$tan(a)\cdot(x-250m)$$

With this function, the left side of the subsiding part of the roof (at x=250 m) would not subside at all, which is unlikely, so the entire function is also shifted downwards by a somewhat arbitrarily chosen amount of b=-25 m to smooth out the transition to the part of the roof which is not directly affected by the forced displacement. (x>250 m) The resulting term for the displacement varying in x-direction is therefore

(4)

$$f\left( x \right)=\tan\left( a \right)\cdot\left( x-250m \right)+b$$

The dependency of the displacement on y was added to reduce stresses and weaken stress singularities at the contacts of the dyke with the sides of the pluton. The aim was to obtain smaller displacements at the edges of the pluton (at y=-500 m and y=800 m) without reducing the displacement near the centre of the dyke/pluton. As a first-order approximation, we decided to use the shape of a parabola

(5)

$$g'\left( y \right)=c_{1}y^{2}+c_{2}y+c_{3}$$

Using g’(0)=f(x=0)=c’+b=c and g’(-500 m)=g’(800 m)=0 as boundary conditions to determine the three constants, leading to

(6)

$$g'\left( y \right)=\frac{b-c}{400,000m^{2}}y^{2}-0.375\frac{b-c}{500m}y+c$$

This function was scaled so that it is equal to 1 at y=0 (at the Rílutungnahamrar-outcrop):

(7)

$$g\left( y \right)=\frac{g'(y)}{c}=\frac{b-c}{400,000m^{2} c}y^{2}-0.375\frac{b-c}{500m c}y+1$$

Multiplying the two expressions for eq(4) and eq(7) results in the expression used to force the vertical displacement shown in eq(1) in the main text.

Most deformation models, which study magma chambers, apply a symmetrical pressure or volume change around their deformation source. In our model, localized subsidence affects specifically the section of the pluton roof adjacent to the dyke (eq. 1 in the main text) and additional boundary conditions are required for the other boundaries of the magma body. We chose to apply a moderate pressure change of -1MPa to all other boundaries of the cavity, which represents our magma body. The deformation caused by this pressure change may be understood as a reaction to the removal of material from the reservoir, but it is negligible compared to the deformation caused by the forced subsidence of the collapsing part of the roof. However, it prevents the boundaries from caving in (as it would have been the case for unconstrained boundaries) without fixing them entirely in any direction of motion. No constraints were put on the dyke walls other than that they cannot move into each other.

The mesh used for our deformation model was created from tetrahedral elements of varying sizes. At the walls of the pluton and at the dyke the element sizes ranged from 1–50m with an element growth rate of 1.15. A 20 km x 20 km square at the surface centred above the Rílutungnahamrar-outcrop allowed elements to vary between 1 m–100 m with a growth rate of 1.25. Everywhere else in the domain elements were allowed to measure up to 15 km with a growth rate of 1.45. The model consisted of a total of 1175612 elements.

Whenever possible, it is important to benchmark numerical models against a known solution, e.g. an analytical model to ensure that the model does not contain any inherent errors (Hickey and Gottsmann 2014). There is no model or data to directly compare our model setup to, but it can be understood as a combination or superposition of two deformation sources (the magma reservoir and the dyke), for which comparable solutions and implementations can be found, e.g. in the dMODELS-package by Battaglia *et al.* (2013).

Deformation caused by only the dyke was compared to the dMODELS-implementation of a rectangular dislocation source (Battaglia *et al.*, 2013), which is based on Okada (1985). In contrast to the model used in the study, where the dyke opens as a result of the collapsing magma body roof, we applied a uniform opening of 1m across the dyke here. The difference between the two models was up to 4-8% of maximum displacement of the numerical model in each of the directions of motion with a slightly larger deviation in the vertical component compared to the horizontal ones. The numerical model slightly underestimating surface deformation compared to the analytical one.

There is no analytical model for a box-shaped deformation source, but the dimensions of the magma body were adjusted to resemble a pressurized, horizontal, inflating circular crack (Fialko *et al.,* 2001), so that the sill-package of the dMODELS package (Battaglia *et al.,* 2013) could be applied using Matlab R2019a (<https://matlab.mathworks.com/>). It is assumed that changing the dimensions of the magma body does not introduce any errors into the model. The pennyshaped crack had a radius of 1 km and the box-shaped magma-chamber measured 1.77 km x 1.77 km x 10 m. These dimensions were chosen so that the base area of the circular crack and the box were comparable. For both models the centre depth was equal to d=2 km, the pressure change was set to 10 MPa, E=30 GPa and ν=0.25. It should be noted, that the forced displacement applied to the roof in the actual model was not applied during the benchmarking. Results of the benchmarking indicate that despite the varying source geometries, the difference between the two models ranges between 6-8% for each of the three components. The numerical model underestimated the deformation compared to the analytical model, which is to be expected given that the diameter of the crack (2 km) was slightly larger than the length of the sides of the box (1.77 km).

The assumption that there are no initial stresses in the model domain implies lithostatic equilibrium and any stresses given in the model results are to be understood as deviatoric stresses. Consequently, the model does not consider gravity. An attempt to apply gravity to the model instead of the forced displacement (eq. 1) did not reproduce the observed tilt of the pluton roof adjacent to the dyke, so it was not considered in the final model.

## References for Supplementary Material

Battaglia, M., Cervelli, P. F., & Murray, J. R. (2013). dMODELS: A MATLAB software package for modeling crustal deformation near active faults and volcanic centers. *Journal of Volcanology and Geothermal Research*, *254*, 1-4.

Fialko, Y., Khazan, Y., & Simons, M. (2001). Deformation due to a pressurized horizontal circular crack in an elastic half-space, with applications to volcano geodesy. *Geophysical Journal International*, *146*(1), 181-190.

Hickey, J., & Gottsmann, J. (2014). Benchmarking and developing numerical Finite Element models of volcanic deformation. *Journal of Volcanology and Geothermal Research*, *280*, 126-130.

Okada, Y. (1985). Surface deformation due to shear and tensile faults in a half-space. *Bulletin of the seismological society of America*, *75*(4), 1135-1154.
